# Natal habitat and sex-specific survival rates result in a male-biased adult sex ratio

**DOI:** 10.1093/beheco/arz021

**Published:** 2019-02-22

**Authors:** A H Jelle Loonstra, Mo A Verhoeven, Nathan R Senner, Jos C E W Hooijmeijer, Theunis Piersma, Rosemarie Kentie

**Affiliations:** 1 Conservation Ecology Group, Groningen Institute for Evolutionary Life Sciences (GELIFES), University of Groningen, Groningen, The Netherlands; 2 Department of Biological Sciences, University of South Carolina, Columbia, SC, USA; 3 NIOZ Royal Netherlands Institute for Sea Research, Department of Coastal Systems, Utrecht University, Texel, The Netherlands; 4 Department of Zoology, University of Oxford, Oxford, UK

**Keywords:** adult sex ratio, hatching sex ratio, *Limosa limosa limosa*, mark-recapture, sex-specific survival

## Abstract

The adult sex ratio (ASR) is a crucial component of the ecological and evolutionary forces shaping the dynamics of a population. Although in many declining populations ASRs have been reported to be skewed, empirical studies exploring the demographic factors shaping ASRs are still rare. In this study of the socially monogamous and sexually dimorphic Black-tailed Godwit (*Limosa limosa limosa*), we aim to evaluate the sex ratio of chicks at hatch and the subsequent sex-specific survival differences occurring over 3 subsequent life stages. We found that, at hatch, the sex ratio did not deviate from parity. However, the survival of pre-fledged females was 15–30% lower than that of males and the sex bias in survival was higher in low-quality habitat. Additionally, survival of adult females was almost 5% lower than that of adult males. Because survival rates of males and females did not differ during other life-history stages, the ASR in the population was biased toward males. Because females are larger than males, food limitations during development or sex-specific differences in the duration of development may explain the lower survival of female chicks. Differences among adults are less obvious and suggest previously unknown sex-related selection pressures. Irrespective of the underlying causes, by reducing the available number of females in this socially monogamous species, a male-biased ASR is likely to contribute to the ongoing decline of the Dutch godwit population.

## Teaser

Skewed adult sex ratios (ASRs) may affect population dynamics and sexual selection. We report a male-biased ASR in Black-tailed Godwits that results from male-biased survival during the pre-fledging period and adulthood. The relative difference in sex-specific survival during pre-fledging was higher in low-quality habitats. Our study demonstrates how both habitat-dependent and independent demographic processes acting at different life-history stages can influence the number of males and females available to reproduce in a population.

## INTRODUCTION

The ratio of males to females is a crucial characteristic of any population as it likely affects the competition for mates among individuals and, hence, the population’s mating system, dispersal and migratory behavior, and demographics (Bessa-Gomes et al. 2004; Kokko et al. 2006; Trochet et al. 2013; Lisovski et al. 2016; Eberhart-Phillips et al. 2018). The ecological basis of deviations from an equal sex ratio can therefore affect a population’s viability (Wedekind 2002; Donald 2007; Grayson et al. 2014; Morrison et al. 2016; Ramula et al. 2018).

The causes and consequences of variation in the sex ratios of birds have been intensively studied (Weatherhead and Teather 1991; Benito and Gonzales-Solis 2007; Eberhart-Phillips et al. 2017). Thereby, studies show that birds are able to mold the sex ratio of their clutches in response to the condition of the mother, their lay date and hatch order, or the quality of the breeding environment (Clout et al. 2002; Suarsa et al. 2003; Alonso-Alvarez 2006; Dijkstra et al. 2010). However, skewed initial sex ratios are only one potential determinant of the adult sex ratio (ASR), as sex differences in survival during other life-history stages can also contribute to the ASR (Emlen 1997; Weimerskirch et al. 2005; Benito and Gonzales-Solis 2007; Eberhart-Phillips et al. 2017). In a now classic paper, Fisher (1930) predicted that if the costs and benefits of raising offspring of either sex are equal for both parents, sex ratios should be equal at the cessation of parental care. In contrast, if the 2 sexes differ in cost—e.g., in their nutritional needs due to different developmental trajectories because of sexual size dimorphism—the more expensive sex is expected to experience a higher mortality when conditions are limiting (Benito and Gonzales-Solis 2007; Villegas et al. 2013). Additionally, sex-specific reproductive costs during adulthood may cause sex-specific survival rates that potentially introduce a shift in the ASR as well (Tavecchia et al. 2001).

Despite the importance of variation in ASRs to the demography of natural populations (Székely et al. 2014a), studies exploring the entire range of temporal and spatial variation in ASRs within single species are scarce (but see: Kosztolányi et al. 2011; Morrison et al. 2016; Eberhart-Phillips et al. 2017). As a result, there is no consensus on the contribution and causes of the different mechanisms causing variation in ASRs. Furthermore, understanding the ecological correlates of factors shaping an unequal ASR is not only of interest from an ecological and evolutionary perspective, but is especially important to understanding how best to conserve declining and endangered species with skewed sex ratios (Pike and Petrie 2003; Eberhart-Phillips et al. 2018).

Continental Black-tailed Godwits *Limosa limosa limosa* (hereafter, “godwits”), are socially monogamous and sexually dimorphic shorebirds in which females are the larger sex from an early age onwards (Schroeder et al. 2008; Loonstra et al. 2018). Over the past 45 years, the population of godwits breeding in The Netherlands has declined in concert with the steadily intensifying use of their farmland breeding habitat (Kentie et al. 2016). These changes in their breeding habitat have, in particular, affected chick survival (Kentie et al. 2013, 2018). Previous work has also shown that female chicks have lower relative body masses and growth rates in the wild than males, suggesting that the condition of female chicks is constrained more than that of males which can potentially lead to sex-specific mortality rates during this life-history stage (Loonstra et al. 2018).

To investigate whether the ASR of godwits is biased and whether variation in habitat quality could contribute to such a bias, we estimated the sex ratio of godwits at hatch and the sex-specific survival of individually marked godwits during 3 subsequent life-history stages: the pre-fledging chick stage, post-fledging juvenile stage, and adult stage. Fieldwork was conducted in one of the strongholds of the godwit population in southwest Friesland, The Netherlands. Based on previously reported results on the sex-specific condition of godwit chicks (Loonstra et al. 2018), we predicted that only survival during the pre-fledging period would be sex-dependent—with lower survival probabilities for females—but that post-fledging and adult survival would be equal between the sexes. Consequently, we predicted that if pre-fledged females do have a lower survival rate during the period of parental care, we would observe a female-biased sex ratio at hatch (Fisher 1930; Hamilton 1967). A subsequent bias in the ASR would then depend on the balance between the bias in the sex ratio at hatch and that of sex-specific survival rates during the pre-fledging stage. If such a bias exists, it might have significant consequences for the ability of this population to reverse the current negative population growth rate by limiting the reproductive potential of the entire population.

## METHODS

### Study area and population

This study was carried out between 2008 and 2017 and centered at 52°55′N, 5°25′E (Kentie et al. 2018). During this time, the extent of the study area grew from 8.780 (2008–2011) to ~11.495 ha (2012–2017; Senner et al. 2015a). Adult godwits were generally present in the study area from late February until late August. Between early April and early June, godwits laid clutches with an invariant size of 4 eggs (Senner et al. 2015b). Nests were located in a variety of grassland types, ranging from dairy farmland with a high intensity of agricultural land usage (~35% of nests) to less intensely used herb-rich grasslands (~65% of nests; see: Groen et al. 2012). We assigned fields to 1 of 2 classes based on their plant species richness and the presence of foot drains (Groen et al. 2012; Kentie et al. 2013), and used the names “meadows” and “monocultures” to refer to these 2 classes (see: Kentie et al. 2013 for more details). Precocial chicks hatch after an incubation period of approximately 21 days and fledge when c. 25 days old (Kruk et al. 1997). After this period, parents can accompany chicks for another 1–2 weeks (Loonstra AHJ and Verhoeven MA, personal observation), with fledged chicks being present in the study area until late September (Verhoeven MA and Loonstra AHJ, personal observation).

### Data collection

Godwit nests were located by members of our field team, local landowners, and volunteers. Once a nest was found, we used the egg flotation method to estimate lay date and predict hatching date so that the chicks could be ringed before leaving the nest (Liebezeit et al. 2007). From 2008 to 2016, 1-day-old chicks were marked with a plastic flag engraved with a unique alphanumeric code. If we recaptured a chick at an age of 10 days or older, we replaced its engraved flag with a metal ring and unique combination of 4 colored rings and a colored flag. This combination of color rings is easier to see from a distance, but does not fit on the shorter legs of young chicks.

We obtained a 30-μl blood sample by bleeding the leg vein of <15-day-old chicks and the wing vein of older chicks and adults during the ringing process in order to determine the genetic sex of each individual. Blood was stored in individually labeled 1.5-ml Eppendorf tubes containing 95% alcohol buffer and frozen at −80°C as soon as possible. Individuals were then molecularly sexed using methods described by Schroeder et al. (2010).

Both field team members and volunteers reported observations of marked individuals. Individuals were resighted opportunistically throughout the year (e.g., at their wintering location in West Africa or on the Iberian Peninsula, June–April), and we made daily focused efforts during the pre-breeding, breeding, and post-breeding periods in The Netherlands (March–August) and the spring staging period on the Iberian Peninsula (January–March). To avoid the incorporation of misread flag and color-mark combinations—which can bias survival estimates—we removed observations of individuals that were only seen a single time in a season.

### Estimating hatching sex ratio

To determine whether the sex ratio of chicks at hatch significantly deviated from parity, we used a general linear mixed effect model with a binomial error structure and a logit function with the sex of the chick as the response variable in the package “lme4” (Bates et al. 2015) in Program R (v. 3.4.3; R Core Development Team 2017). To prevent mixing of chicks from different nests, we only used nests of which all 4 chicks were present during ringing on the actual hatch day. Year (2008–2016) and natal habitat type were included in the model as factors to determine whether sex ratios differed among years or habitat type. To assess whether the sex ratio at hatch varied during the breeding season, we included a nest’s hatch date (relative to the annual mean hatch date) as a continuous covariate. All models contained “NestID” as a random effect in order to control for the nonindependence of chicks from the same nest. To test the significance of each covariate, we followed a stepwise backward procedure in which we deleted terms in order of decreasing significance and tested the influence of the intercept on its significance with α = 0.05 (Quinn and Keough 2005).

### Mark-recapture survival analysis

**Figure 1:**
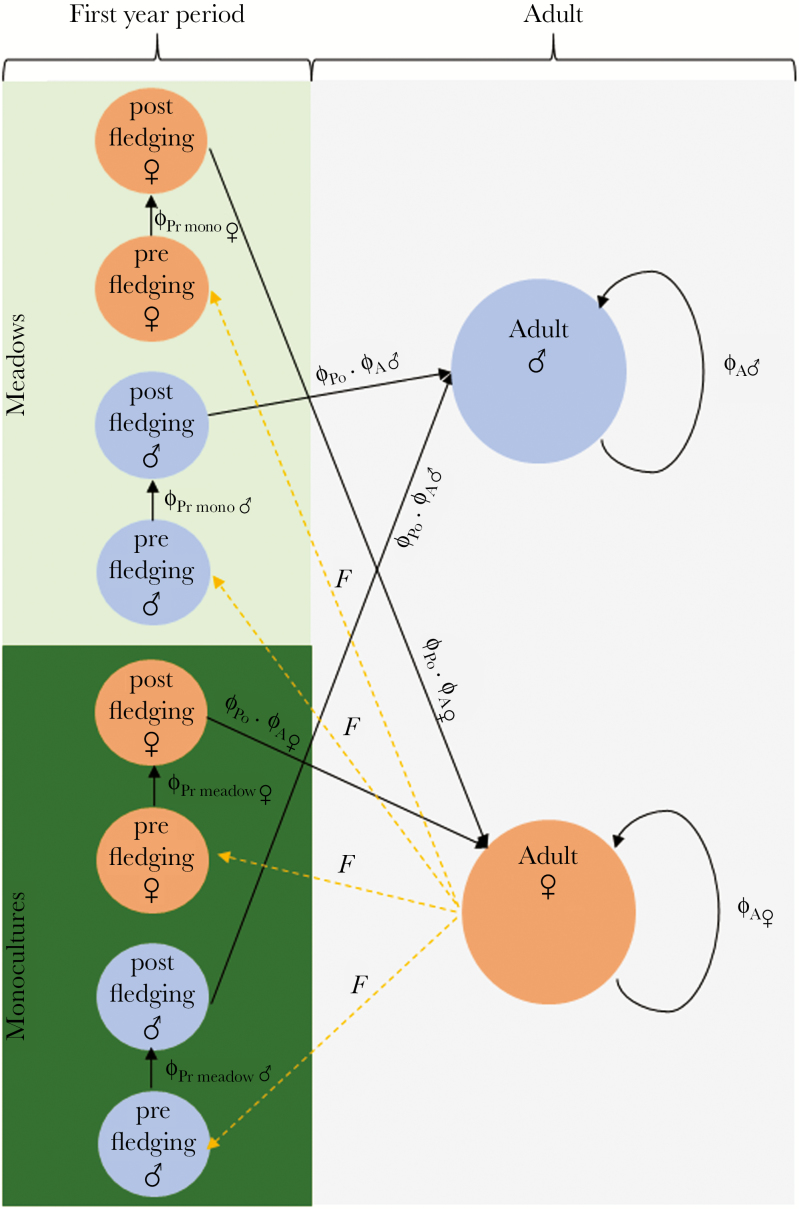
Godwit lifecycle flow diagram illustrating survival rates (ɸ) among the 3 studied life stages (Pre-fledging = Pr, Post-fledging = Po, and adult = A) at the 2 different habitats (monocultures = mono and herb-rich meadows = meadow). Solid black lines represent the different survival rates between or within life stages and the dashed yellow line the fecundity (F). Fecundity is expressed as the number of adult females (n♀), assuming a modal clutch size of 4 eggs (k), a habitat-dependent nest survival rate (HDNS) and a habitat-dependent nest distribution (HDND): F = n♀ · k · HDD · HDNS.

We used observations of all chicks that were marked when 1-day-old from 2008 to 2016 to create encounter histories for each individual. Our final dataset consisted of 4390 individuals (2097 males, 2293 females; Table 1). We used Cormack-Jolly-Seber models to estimate sex-specific apparent survival (Cormack 1964; Jolly 1965; Seber 1965). We considered 3 different age classes: ɸ_pre-fledging_, ɸ_post-fledging_, and ɸ_adult_ (Figure 1). The length of the first period was defined as the mean interval between hatching and the first sighting of all individuals that were seen after fledging on the breeding grounds in post-breeding groups (ɸ_pre-fledging_ = 45 ± 11 days). For pre-fledged chicks, we also tested for effects of natal habitat (monoculture or meadow) and year on survival (Kentie et al. 2013). The post-fledging period lasted 320 days. Apparent adult survival (ɸ_adult_) estimates were modeled over 1-year time intervals. Due to the small sample size of individuals that entered the post-fledging period in some years, we were unable to include a year effect on post-fledging and adult survival in our models.

**Table 1:** Total number of complete clutches per year used for the analysis of sex ratios at hatch and the number of 1-day-old godwit chicks marked from 2008 to 2016 during the breeding season in southwest Friesland, The Netherlands, by sex, habitat type—monoculture or meadow—and year

Year	Total number of complete clutches	Sex	Monoculture	Meadow
2008	10	Males	31	95
		Females	37	92
2009	8	Males	27	105
		Females	34	100
2010	23	Males	55	132
		Females	61	158
2011	5	Males	11	39
		Females	10	48
2012	41	Males	78	153
		Females	85	197
2013	54	Males	119	300
		Females	104	347
2014	26	Males	50	218
		Females	58	253
2015	34	Males	84	184
		Females	84	213
2016	92	Males	141	275
		Females	109	303
Total	293	Males	596	1.501
		Females	582	1.711

A preliminary inspection of our data revealed differences in the resighting probability among all age categories. This was most likely because the majority of resightings were made on the breeding grounds and are thus sensitive to behavioral differences between age classes. For this reason, we allowed resighting probability to vary with age. Additionally, our resighting effort varied over the years; all classes thus include year (y) as a covariate of the resighting probability. We also included sex (s) as a covariate of the resighting probability for all 3 age classes (Figure 1). In doing so, we accounted for potential differences in behavior between males and females that could result in sex-specific detection probabilities (Amrhein et al. 2012). Finally, to account for differences in the resighting probability of individuals with different marking schemes (e.g., engraved flags vs. full color-ring combinations), all models included an effect of ringtype (ring) on the resighting probability.

Because of the number of parameters involved, we performed a stepwise model selection procedure (Doherty et al. 2012). First, we selected an a priori set of candidate models for the resighting probability (*P*) for the 3 age classes (Table 2, Supplementary Table S1). During this first step, we defined the most parsimonious model for *P*, but the survival probability during the different age categories was modeled as in the full model (ɸ_pre-fleding·sex·habitat·y_ + ɸ_post-fledging·sex_ + ɸ_adult·sex_). Second, we used the most parsimonious parameterization of *P* to investigate the most parsimonious parameterization of the models describing the survival probability among the different age classes (Table 3, Supplementary Table S2).

**Table 2:** Model selection results for the first 5 competing resighting probability models (*P*), step 1

Parameterization of *P*		*K*	Δ QAIC_c_	Model weight	Δ Qdev
1)	*P*_Pre-fledging·y + Pre-fledging·s + Post-fledging·y + Adult·s·y_	75	0.00^a^	0.73	4.88
2)	*P*_Pre-fledging·y + Post-fledging·y + Adult·s·y_	74	3.12	0.25	9.05
3)	*P*_Pre-fledging·y + Pre-fledging·s + Post-fledging·y·s + Adult·s + Adult·y_	73	9.41	0.01	18.40
4)	*P*_Pre-fledging·y + Post-fledging·y·s + Adult·s·y_	82	9.49	0.01	0.00^b^
5)	*P*_Pre-fledging·y + Pre-fledging·s + Post-fledging·y + Post-fledging·s + Adult·s·y_	72	9.82	0.01	20.85

For all models we modeled the survival probability as in the full model: (Φ_Pre-fledging_·s_HT_·y + Φ_Post-fledging_·s + Φ_Adult_·s). Each model contained an effect of ring type. Model selection results for all tested models can be found in Supplementary Table S1. *P*_Pre-fledging_ = resighting probability from hatch till fledge; *P*_Post-fledging_ = Resighting probability from post-fledging till first adult period; *P*_Adult_ = resighting probability during adulthood; s = molecular sex; y = year. “∙” indicates an interaction between effects; *K* = number of parameters; Δ Qdev = the QDeviance relative to that of the best fitting model (with the lowest QDeviance); Δ QAICc = QAICc relative to the best-supported model (with the lowest QAICc).

^a^QAIC_c_ = 6779.32.

^b^QDev = 1462.56.

**Table 3:** Model selection results for the first 5 competing apparent survival probability (Φ) models during all 3 life stages (pre-fledging, post-fledging and adult; step 2)

Parameterization of Φ		*K*	Δ QAIC_c_	Model weight	Δ Qdev
1)	Φ_Pre-fledging·y + Pre-fledging·HT·s + Post-fledging + Adult·s_	51	0.00^a^	0.88	44.93
2)	Φ_Pre-fledging·HT·s·y + Post-fledging·s + Adult·s_	75	4.11	0.11	0.00^b^
3)	Φ_Pre-fledging·s + Pre-fledging·HT·y + Post-fledging + Adult_	56	14.59	0.00	49.33
4)	Φ_Pre-fledging·s + Pre-fledging·HT·y + Post-fledging + Adult·s_	57	15.26	0.00	47.96
5)	Φ_Pre-fledging·s + Pre-fledging·HT·y + Post-fledging·s + Adult·s_	58	17.18	0.00	47.85

For all models we modeled the resighting probability as in the best-supported model of step 1: (*P*_Pre-fledging·y + Pre-fledging·s + Post-fledging·y + Adult·s·y + ringtype_). Model selection results for all tested models can be found in Supplementary Table S2. Φ_Pre-fledging_ = apparent survival probability during the pre-fledging period; Φ_Post-fledging_ = apparent survival probability during post-fledging period; Φ_Adult_ = apparent survival probability of adults; HT = natal habitat type type, monoculture vs. herb-rich meadow; s = molecular sex; y = year. “∙” indicates an interaction between effects; *K* = number of parameters; Δ Qdev = the QDeviance relative to that of the best fitting model (with the lowest QDeviance); Δ QAICc = QAICc relative to the best-supported model (with the lowest QAICc).

^a^QAIC_c_ = 6775.21.

^b^QDev = 1467.45.

All mark-recapture models were constructed using the package “Rmark” (Laake 2013) and run with the program “MARK” (White and Burnham 1999). The goodness-of-fit (GOF) for the global model was assessed using the median ĉ-hat test (100 iterations) in Program MARK (White and Burnham 1999). Because the data were slightly overdispersed (ĉ = 1.25 ± 0.01), we used QAICc (Akaike’s information criterion, corrected for overdispersion and small sample size) for model interpretation and evaluation (Burnham and Anderson 2002). Model selection was based on Akaike’s Information Criterion scores adjusted for small sample sizes (AIC_c_); models differing by <2 AIC_c_ units and without uninformative parameters were considered the most parsimonious model (Arnold 2010). All reported confidence intervals were adjusted for overdispersion.

### Estimating ASR

To estimate the ASR, we applied a 2-sex matrix that incorporates all life stages into 2 age classes: first-year and adults (Figure 1). We allowed adults of both sexes to disperse between the 2 habitats so that the distribution of godwits during each time step between the 2 habitats resembled the distribution of nests in our study area over the entire study period (33% monocultures and 67% meadows). We assumed a clutch size of 4 eggs with an unbiased sex ratio and a daily nest survival of 0.962 for nests laid in monocultures and 0.973 for nests laid in meadows (Kentie et al. 2015). Furthermore, we assumed that males and females become sexually active at an age of 2. To parameterize the model, we used our own calculated life-stage dependent survival estimates.

To identify during which life-history stage differences in survival rates between the sexes had the largest effect on the ASR, we calculated the ASR using a stable age distribution in a hypothetical 2-sex matrix in which the survival rates of the 2 sexes were equivalent in all life stages except the stage of interest. By doing so, we could separately determine the effect of each sex-dependent life-history stage on the ASR.

## RESULTS

Sex ratio at hatching among all 293 complete nests was on average 48.4% males, which did not deviate from parity (*P* = 0.27; Table 4). In addition, we did not find any association between sex ratio at hatch and natal habitat type, relative hatch date, or year (Table 4).

**Table 4:** Results of a generalized linear mixed model examining the effect of relative hatch date, habitat type—monoculture or meadow—and year on the sex ratio at hatch (0 = male; 1 = female)

Response variable	Fixed effects	Estimate	SE	*P*
Sex ratio	Intercept	0.07	0.06	0.27
	Habitat type^a^	0.13	0.14	0.35
	Relative hatch date	0.0047	0.0075	0.53
	Year 2009^b^	−0.15	0.48	0.75
	Year 2010	0.73	0.38	0.06
	Year 2011	0.30	0.55	0.58
	Year 2012	0.39	0.35	0.26
	Year 2013	0.14	0.34	0.69
	Year 2014	0.18	0.37	0.63
	Year 2015	0.04	0.36	0.91
	Year 2016	0.02	0.33	0.94

Estimates of nonsignificant terms are from the last model before simplification.

^a^Reference level for natal habitat type is “monoculture”.

^b^Reference level for year is 2008.

**Figure 2:**
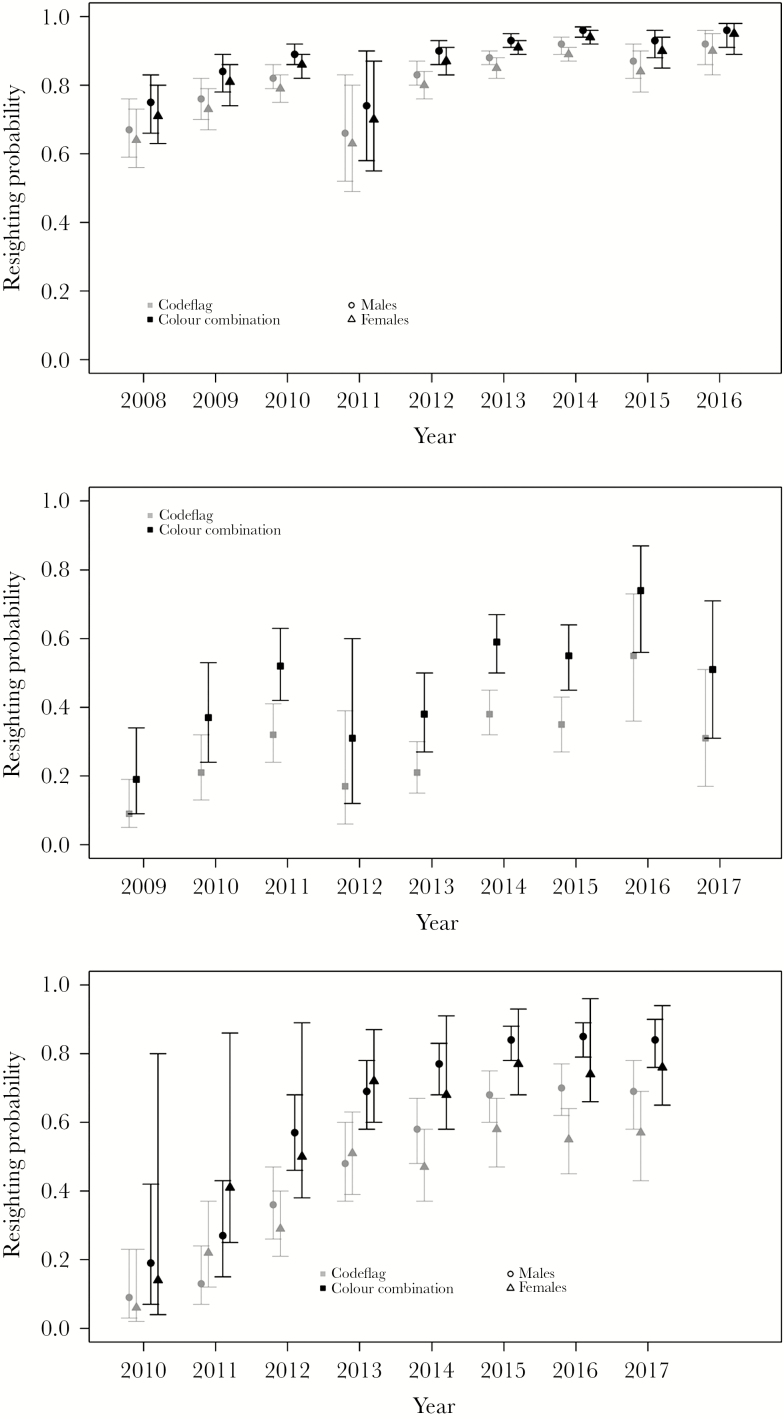
Resighting probabilities of godwits from 2008 to 2017 for the (a) pre-fledging period, (b) post-fledging period and (c) adulthood. Estimates are based on model 1 (Supplementary Table S1).

In our mark-recapture analysis, the most parsimonious model for resighting probability included an effect of year and sex during the pre-fledging period (model 1; Table 2; Supplementary Tables S1 and S3; Figure 2a), a year effect during the post-fledging period (model 1; Table 2; Supplementary Tables S1, S3; Figure 2b), and an interaction term between year and sex for adults (model 1; Table 2; Supplementary Tables S1 and S3; Figure 2c). For all 3 life stages, the resighting probability slightly increased over the course of the study and, in general, females had lower resighting probabilities both as chicks and adults (Supplementary Table S3; Figure 2a–c). This increase in resighting probability is most likely the result of an increase in observation effort as our field team became larger, whereas the low resighting probability of first-year birds is likely due to the fact that a portion of first-year birds remains at nonbreeding sites in Africa throughout the year.

**Figure 3:**
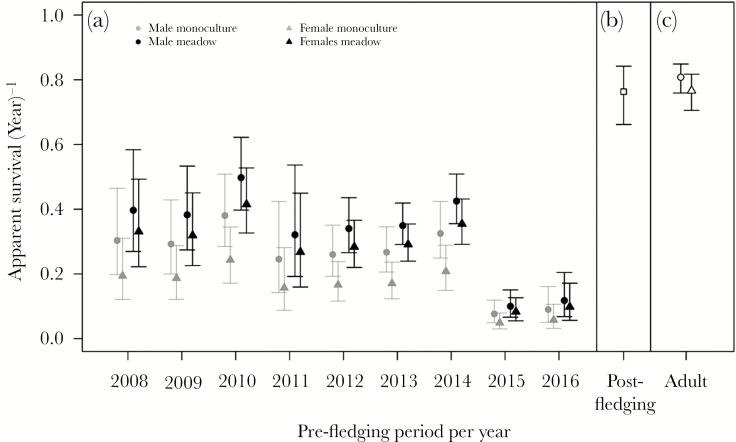
Apparent annual survival estimates of godwits from 2008 to 2016 during the pre-fledging period (a), post-fledging period (b), and adulthood (c). Estimates are based on model 1 (Supplementary Table S2).

Apparent pre-fledging survival probability was lower for females than for males, was lower on monocultures than on meadows, and sex difference in survival was strongest on monocultures (ΔAIC_c_ = 4.11; Tables 3 and 5; Supplementary Table S2; Figure 3a). Apparent survival of males during the pre-fledging period ranged between years and habitats from 0.08 to 0.50, and for females from 0.05 to 0.42 (Table 5). The sex bias (ɸ_♂-chick_/(ɸ_♂-chick_ + ɸ_♀-chick_)) in apparent survival was higher in monocultures (0.61, 95% CI = 0.43–0.77) than in meadows (0.55, 95% CI = 0.41–0.69). The estimates of apparent survival during the post-fledging period did not differ between the sexes (ɸ = 0.76, 95% CI = 0.71–0.81; Supplementary Table S2, Figure 3b), but adult females had lower survival rates than males (ɸ males = 0.81, 95% CI = 0.76–0.84; ɸ females = 0.77, 95% CI = 0.71–0.82; Supplementary Table S2, Figure 3c), although their confidence intervals were overlapping.

**Table 5:** Estimates and 95% confidence intervals of annual apparent survival during the pre-fledging period, for both sexes and habitat types

Year	Male monoculture	Female monoculture	Male meadow	Female meadow
2008	0.30 (0.20–0.46)	0.19 (0.12–0.31)	0.40 (0.27–0.58)	0.33 (0.22–0.49)
2009	0.29 (0.20–0.43)	0.19 (0.12–0.29)	0.38 (0.27–0.53)	0.32 (0.23–0.45)
2010	0.38 (0.29–0.51)	0.24 (0.17–0.34)	0.50 (0.37–0.62)	0.42 (0.33–0.53)
2011	0.25 (0.14–0.42)	0.16 (0.09–0.28)	0.32 (0.19–0.54)	0.27 (0.16–0.45)
2012	0.26 (0.19–0.35)	0.17 (0.12–0.24)	0.34 (0.27–0.44)	0.28 (0.22–0.37)
2013	0.27 (0.21–0.35)	0.17 (0.12–0.24)	0.35 (0.29–0.42)	0.29 (0.24–0.35)
2014	0.32 (0.25–0.42)	0.21 (0.15–0.29)	0.43 (0.36–0.51)	0.35 (0.29–0.43)
2015	0.08 (0.05–0.12)	0.05 (0.03–0.08)	0.10 (0.07–0.15)	0.08 (0.05–0.14)
2016	0.09 (0.05–0.16)	0.06 (0.03–0.10)	0.12 (0.07–0.20)	0.10 (0.06–0.17)

Estimates are based on model 1 (Table 3).

Differences in sex-specific survival rates during both the pre-fledging and adult periods resulted in a male-biased ASR. The ASR modeled under a stable age distribution and expressed as the proportion of males was 0.64. The sex difference in survival during the adult period had the largest effect on the ASR (ASR: 0.58 adult period alone vs. ASR: 0.55 chick period alone). During the pre-fledging period, the sex-specific survival component of chicks hatched on meadows (0.53) had a slightly higher impact on the ASR than that of chicks hatched on monocultures (0.52).

## DISCUSSION

We evaluated the sex ratio at hatch and sex-specific survival rates of Continental Black-tailed Godwits during 3 life-history stages to assess if their ASR was skewed and, if so, when this skew arose. We found that the sex ratio at hatch was at parity, but that lower survival rates of females during the pre-fledging and during adulthood resulted in a male-biased ASR. Our results are in line with the notion that ASRs are frequently unequal and male-biased in nature (Donald 2007; Székely et al. 2014a, 2014b). This male-biased ASR, in turn, may limit the ability of godwits to reverse their ongoing decline by forcing males to remain unpaired throughout the breeding season.

### Causes of variation in ASR

We did not find a bias in the sex ratio of godwits at hatch, which appears in contradiction with theoretical predictions (Fisher 1930; Hamilton 1967). However, our defined pre-fledging phase (45 days) already covers part of the post-fledging phase, as most chicks like fledge before an age of 45 days. The sex-specific mortality rates that we observed could therefore still result from mortality events occurring after the cessation of parental care. For this reason, we cannot conclusively reject the prediction that differences in mortality between sexes during the period of parental care should be offset by a skewed sex ratio at hatch (Fisher 1930; Hamilton 1967).

However, as we predicted based on sex-dependent differences in the condition of chicks (Loonstra et al. 2018), we did find an effect of sex on the apparent survival probability of godwit chicks during the pre-fledging period. Furthermore, we also found an interaction between natal habitat type and sex on apparent survival during this period, with the relative skew in sex-specific survival being larger in monocultures (the habitat type with general lower survival rates, i.e., lower quality habitat). This suggests 2 things: First, that the relatively lower body condition of female chicks in comparison with males (Loonstra et al. 2018) either directly causes increased mortality rates among females or that female development (e.g., time-to-fledging) is delayed and causes an increased vulnerability to predation. Second, the lower body condition of female chicks appears to relate to habitat-specific characteristics that differentially affect males and females. This is not altogether surprising: because females are the larger sex from an early age onwards and thus need more energy during development (Loonstra et al. 2018). Lower food availability on monocultures (Schekkerman and Beintema 2007) could therefore affect females disproportionally (Loonstra et al. 2018). However, before we can determine the causal relationship between differences in habitat- and sex-specific survival, we need studies that not only follow the larger-scale movements of chicks over time, but also determine the exact cause of their deaths (Schekkerman et al. 2009).

During adulthood, we also found that males and females differed in their survival rates. The underlying causes of these sex-specific differences are unclear. However, we suspect that this difference most likely arises during northbound migration during flights over the Sahara desert and/or on the breeding grounds (Senner et al. in review). For instance, it could be that because females are larger, they experience a higher mortality during migration, as they need more nutrients to refuel. Alternatively, due to their larger size, females could be less agile and more vulnerable to predation at staging and breeding sites (Post and Götmark 2006). It is also possible that during the breeding season, females and males have different incubation patterns (Bulla et al. 2016) and that these different incubation schedules result in differences in survival (Arnold et al. 2012). Finally, because more males survive to adulthood, a smaller proportion of males will be involved in incubation and chick-rearing than females. Thus, if there are direct costs of these reproductive activities (e.g., predation) and/or energetic costs stemming from them that carry over to affect survival via reversible state effects (Senner et al. 2015c), surviving females may disproportionately suffer the consequences and have reduced survival rates during adulthood.

### Changing ASR in godwits

If we assume 1) no sex-specific immigration or emigration into, or out of, our local study population, and 2) that we followed a representative distribution of nests and accurately measured nest survival in the 2 habitat types, our results predict a strongly male-biased population that is mostly driven by the sex-specific survival rates of adults. Nonetheless, it is important to realize that the distribution of nests among the 2 habitat types is unbalanced and varies among years—33% of nests occur in monocultures and 67% in meadows (Kentie et al. 2015)—as do habitat specific nest survival rates. Nests experience an average daily nest survival rate of 0.962 in monocultures compared to 0.973 in meadows (Kentie et al. 2015). As a result, the yearly change in ASR will strongly depend on the breeding distribution of godwits across these 2 habitat types and the annual variation in both nest and chick survival of the godwits.

### Caveats in studies of ASR

Given the importance of ASRs to ecology and evolution, it is widely acknowledged that there needs to be a better understanding of the causes underlying biases in ASR (e.g., Székely et al. 2014b). However, obtaining robust estimates of ASRs are challenging and one of the main reasons why we lack such information about most species. We fully recognize that our estimates could be biased for a number of reasons. For example, our sex ratios at hatch are based on nests in which all chicks hatched; however, if hatching is sex-specific our sex ratios at hatch might be biased (Eiby et al. 2008). Similarly, our estimates of apparent survival might be confounded by permanent sex-dependent emigration from our study area (Julliard 2000; Amrhein et al. 2012). Nonetheless, we believe our survival estimates are robust because: 1) our resightings of marked godwits not only came from the breeding grounds, but also from several known staging and winter sites (Kentie et al. 2016) and 2) previous work by Kentie et al. (2014) did not find an effect of sex on natal dispersal, indicating that our sex-dependent resighting probabilities are likely not caused by a higher dispersal rate among females. However, our adult survival estimates are somewhat lower than those estimated by Kentie et al. (2016), but comparable to those of van Noordwijk and Thomson (2008). The lower survival estimates we report, though, are likely the result of the fact that we, unlike van Noordwijk and Thomson (2008) and Kentie et al. (2016), included the first northbound migration of young godwits in our analyses, an event that is likely to be more dangerous than subsequent bouts of migration (Sergio et al. 2014).

### Implications of bias in ASR

Our results raise questions about the current viability of the Dutch-breeding population and the potential for godwits to adapt their mating system to contemporary environmental conditions (Eberhart-Phillips et al. 2017, 2018). The current population of godwits breeding in agricultural habitats in The Netherlands is under strong pressure from ongoing agricultural intensification (Kentie et al. 2013, 2018), resulting in an annual population decline of almost 6% over the past decade (Kentie et al. 2016). In addition to this rapid decline, socially monogamous godwits must now also cope with a surplus of males, meaning that fewer godwits are able to find a mate and breed than would be possible in a population with a less biased ASR. Furthermore, it is unlikely that a surplus of females from other populations will be able to immigrate into our study population, as most surrounding landscapes consist of similar or even higher percentages of intensified agricultural land, and thus the sex-specific survival differences that we have identified in southwest Friesland are likely to be pervasive across the godwit breeding range in The Netherlands. Although recent work has revealed a link between ASR and mating system and the growth rate of a population (Eberhart-Phillips et al. 2017)—which would suggest that if godwits have the ability to exhibit a more flexible mating system their population growth rate might be less negatively affected—our own observations do not indicate that godwits will be able to exhibit a different mating strategy in the short term (Verhoeven et al. in preparation).

What is more, our sex-biased survival estimates are in line with similar biases in several other populations in which the survival of the larger sex is substantially lower than that of the smaller sex (Grayson et al. 2014; Morrison et al. 2016). Our results additionally indicate that this discrepancy in the survival of the 2 sexes during the pre-fledging period was more pronounced in habitats characterized by more intensive agricultural practices (Groen et al. 2012). While we can only speculate on the exact causes of this discrepancy, our example demonstrates that declines in breeding habitat quality can directly affect not only the survival rate of a species in general, but also incur sex-specific demographic changes that can potentially affect the growth rate of a population.

## FUNDING

This work was supported by the former Ministry of Agriculture, Nature Management and Food Safety and the Ministry of Economic Affairs; the Province of Fryslân and the Spinoza Premium 2014 awarded to TP by NWO Netherlands Organization for Scientific Research. Additional financial support came from the NWO-TOP grant “Shorebirds in space” awarded to TP, Prins Bernard Cultuurfonds, the Van der Hucht de Beukelaar Stichting, the University of Groningen, Birdlife-Netherlands and WWF-Netherlands through Global Flyway Network and the Chair in Flyway Ecology. RK is funded by the Royal Society.

## 

Data accessibility: Analyses reported in this article can be reproduced using the data provided by Loonstra et al. (2019).

## Supplementary Material

arz021_suppl_Supplementary_Material
