# Supplementary material for: Natal habitat and sex-specific survival rates result in a male-biased adult sex ratio
Source: Behav Ecol. 2019 Feb 22;30(3):843–51. doi: 10.1093/beheco/arz021 (PMC6562303; doi:10.1093/beheco/arz021)
Supplement: arz021_suppl_Supplementary_Material [file arz021_suppl_supplementary_material.docx]

Table S1. Model selection results for the competing resighting probability models (*p*), step 1. For all models we modelled the survival probability as in the full model: (Φ_Pre-fledging_·s·_HT_·y + Φ_Post-fledging_·s + Φ_Adult_·s). Each model contained an effect of ring type.

| Parameterization of p | | *K* | Δ QAIC_c_ | Model weight | Δ Qdev |
| --- | --- | --- | --- | --- | --- |
| 1) | P_Pre-fledging·y + Pre-fledging·s + Post-fledging·y + Adult·s·y_ | 75 | 0.00^1^ | 0.73 | 4.88 |
| 2) | P_Pre-fledging·y + Post-fledging·y + Adult·s·y_ | 74 | 3.12 | 0.25 | 9.05 |
| 3) | P_Pre-fledging·y + Pre-fledging·s + Post-fledging·y·s + Adult·s + Adult·y_ | 73 | 9.41 | 0.01 | 18.40 |
| 4) | P_Pre-fledging·y + Post-fledging·y·s + Adult·s·y_ | 82 | 9.49 | 0.01 | 0.00^2^ |
| 5) | P_Pre-fledging·y + Pre-fledging·s + Post-fledging·y + Post-fledging·s + Adult·s·y_ | 72 | 9.82 | 0.01 | 20.85 |
| 6) | P_Pre-fledging·y·s + Post-fledging·y + Post-fledging·s + Adult·s·y_ | 80 | 15.02 | 0.00 | 9.64 |
| 7) | P_Pre-fledging·y + Post-fledging·y + Post-fledging·s + Adult·s·y_ | 73 | 15.17 | 0.00 | 24.16 |
| 8) | P_Pre-fledging·y + Pre-fledging·s + Post-fledging·y·s + Adult·s·y_ | 80 | 20.42 | 0.00 | 15.04 |
| 9) | P_Pre-fledging·y·s + Post-fledging·y·s + Adult·s·y_ | 89 | 28.28 | 0.00 | 4.38 |
| 10) | P_Pre-fledging·y + Pre-fledging·s + Post-fledging·y·s + Adult·y_ | 70 | 30.89 | 0.00 | 46.02 |
| 11) | P_Pre-fledging·y·s + Post-fledging·y + Adult·s·y_ | 79 | 32.54 | 0.00 | 29.21 |
| 12) | P_Pre-fledging·y·s + Post-fledging·y·s + Adult·s + Adult·y_ | 79 | 62.22 | 0.00 | 58.89 |
| 13) | P_Pre-fledging·y + Post-fledging·y + Post-fledging·s + Adult·y_ | 64 | 78.17 | 0.00 | 105.58 |
| 14) | P_Pre-fledging·y + Pre-fledging·s + Post-fledging·y + Post-fledging·s + Adult·s + Adult·y_ | 66 | 85.49 | 0.00 | 108.81 |
| 15) | P_Pre-fledging·y·s + Post-fledging·y·s + Adult·y_ | 79 | 86.83 | 0.00 | 83.51 |
| 16) | P_Pre-fledging·y + Pre-fledging·s + Post-fledging·y + Adult·s + Adult·y_ | 65 | 89.64 | 0.00 | 115.00 |
| 17) | P_Pre-fledging·y + Post-fledging·y·s + Adult·s + Adult·y_ | 72 | 98.53 | 0.00 | 109.56 |
| 18) | P_Pre-fledging·y + Post-fledging·y + Post-fledging·s + Adult·s + Adult·y_ | 63 | 111.48 | 0.00 | 140.93 |
| 19) | P_Pre-fledging·y·s + Post-fledging·y + Adult·y_ | 68 | 116.37 | 0.00 | 135.60 |
| 20) | P_Pre-fledging·y·s + Post-fledging·y + Post-fledging·s + Adult·s + Adult·y_ | 67 | 119.81 | 0.00 | 141.08 |
| 21) | P_Pre-fledging·y + Post-fledging·y + Adult·y_ | 62 | 121.53 | 0.00 | 153.02 |
| 22) | P_Pre-fledging·y + Post-fledging·y·s + Adult·y_ | 69 | 122.32 | 0.00 | 139.50 |
| 23) | P_Pre-fledging·y + Post-fledging·y + Adult·s + Adult·y_ | 59 | 132.61 | 0.00 | 170.22 |
| 24) | P_Pre-fledging·y·s + Post-fledging·y + Post-fledging·s + Adult·y_ | 69 | 147.00 | 0.00 | 164.18 |
| 25) | P_Pre-fledging·y + Pre-fledging·s + Post-fledging·y + Post-fledging·s + Adult·y_ | 59 | 148.99 | 0.00 | 186.61 |
| 26) | P_Pre-fledging·y + Pre-fledging·s + Post-fledging·y + Adult·y_ | 60 | 178.86 | 0.00 | 214.44 |
| 27) | P_Pre-fledging·y·s + Post-fledging·y + Adult·s + Adult·y_ | 66 | 195.05 | 0.00 | 218.37 |
| P_Pre-fledging_ = resighting probability from hatch till fledge; P_Post-fledging_ = Resighting probability from post-fledging till first adult period; P_Adult_ = resighting probability during adulthood; s = molecular sex; y = year. “∙” indicates an interaction between effects; *K* = number of parameters; Δ Qdev = the QDeviance relative to that of the best fitting model (with the lowest QDeviance); Δ QAICc = QAICc relative to the best-supported model (with the lowest QAICc).  ^1^ QAIC_c_ = 6779,32  ^2^ QDev = 1462,56 | | | | | |

Table S2. Model selection results for the competing apparent survival probability (Φ) models during all three life-stages (pre-fledging, post-fledging and adult; step 2). For all models we modelled the resighting probability as in the best-supported model of step 1: (P_Pre-fledging·y + Pre-fledging·s + Post-fledging·y + Adult·s·y + ringtype_).

| Parameterization of Φ | | *K* | Δ QAIC_c_ | Model weight | Δ Qdev |
| --- | --- | --- | --- | --- | --- |
| 1) | Φ_Pre-fledging·y + Pre-fledging·HT·s + Post-fledging + Adult·s_ | 51 | 0.00 | 0.88 | 44.93 |
| 2) | Φ_Pre-fledging·HT·s·y + Post-fledging·s + Adult·s_ | 75 | 4.11 | 0.11 | 0.00 |
| 3) | Φ_Pre-fledging·s + Pre-fledging·HT·y + Post-fledging + Adult_ | 56 | 14.59 | 0.00 | 49.33 |
| 4) | Φ_Pre-fledging·s + Pre-fledging·HT·y + Post-fledging + Adult·s_ | 57 | 15.26 | 0.00 | 47.96 |
| 5) | Φ_Pre-fledging·s + Pre-fledging·HT·y + Post-fledging·s + Adult·s_ | 58 | 17.18 | 0.00 | 47.85 |
| 6) | Φ_Pre-fledging·HT·y + Post-fledging·s + Adult·s_ | 57 | 18.15 | 0.00 | 50.86 |
| 7) | Φ_Pre-fledging·HT·s·y + Post-fledging + Adult_ | 71 | 18.91 | 0.00 | 23.01 |
| 8) | Φ_Pre-fledging·HT·s·y + Post-fledging + Adult·s_ | 72 | 19.52 | 0.00 | 21.56 |
| 9) | Φ_Pre-fledging·HT·y + Post-fledging + Adult_ | 55 | 22.92 | 0.00 | 59.70 |
| 10) | Φ_Pre-fledging·HT + Pre-fledging·s·y + Post-fledging + Adult_ | 56 | 25.41 | 0.00 | 60.15 |
| 11) | Φ_Pre-fledging·s + Pre-fledging·y + Pre-fledging·HT + Post-fledging + Adult_ | 48 | 26.14 | 0.00 | 77.16 |
| 12) | Φ_Pre-fledging·y + Pre-fledging·HT·s + Post-fledging + Adult_ | 50 | 26.42 | 0.00 | 75.41 |
| 13) | Φ_Pre-fledging·s + Pre-fledging·y + Pre-fledging·HT + Post-fledging + Adult·s_ | 49 | 26.71 | 0.00 | 75.71 |
| 14) | Φ_Pre-fledging·s + Pre-fledging·y + Pre-fledging·HT + Post-fledging·s + Adult_ | 49 | 27.75 | 0.00 | 76.74 |
| 15) | Φ_Pre-fledging·y + Pre-fledging·HT·s + Post-fledging·s + Adult_ | 51 | 28.05 | 0.00 | 75.01 |
| 16) | Φ_Pre-fledging·s + Pre-fledging·y + Pre-fledging·HT + Post-fledging·s + Adult·s_ | 50 | 28.60 | 0.00 | 75.56 |
| 17) | Φ_Pre-fledging·y + Pre-fledging·HT·s + Post-fledging·s + Adult·s_ | 51 | 28.93 | 0.00 | 73.86 |
| 18) | Φ_Pre-fledging·y + Pre-fledging·HT + Post-fledging·s + Adult_ | 48 | 29.12 | 0.00 | 80.15 |
| 19) | Φ_Pre-fledging·y + Pre-fledging·HT + Post-fledging·s + Adult·s_ | 49 | 29.69 | 0.00 | 78.69 |
| 20) | Φ_Pre-fledging·y + Pre-fledging·HT + Post-fledging + Adult_ | 47 | 35.10 | 0.00 | 88.16 |
| 21) | Φ_Pre-fledging·HT·s·y + Post-fledging·s + Adult_ | 68 | 40.93 | 0.00 | 51.17 |
| 22) | Φ_Pre-fledging·HT + Pre-fledging·s·y + Post-fledging + Adult·s_ | 56 | 54.47 | 0.00 | 89.22 |
| 23) | Φ_Pre-fledging·HT·y + Post-fledging + Adult·s_ | 54 | 69.46 | 0.00 | 108.27 |
| 24) | Φ_Pre-fledging·y + Pre-fledging·HT + Post-fledging + Adult·s_ | 49 | 169.32 | 0.00 | 218.31 |
| 25) | Φ_Pre-fledging·HT + Pre-fledging·s·y + Post-fledging·s + Adult·s_ | 58 | 171.07 | 0.00 | 201.73 |
| 26) | Φ_Pre-fledging·HT·y + Post-fledging·s + Adult_ | 55 | 177.57 | 0.00 | 214.35 |
| 27) | Φ_Pre-fledging·s + Pre-fledging·HT·y + Post-fledging·s + Adult_ | 54 | 188.63 | 0.00 | 227.45 |
| 28) | Φ_Pre-fledging·HT + Pre-fledging·s·y + Post-fledging·s + Adult_ | 56 | 196.20 | 0.00 | 230.94 |
| Φ_Pre-fledging_ = apparent survival probability during the pre-fledging period; Φ_Post-fledging_ = apparent survival probability during post-fledging period; Φ_Adult_ = apparent survival probability of adults; HT = natal habtat type type, monoculture vs. herb-rich meadow; s = molecular sex; y = year. “∙” indicates an interaction between effects; *K* = number of parameters; Δ Qdev = the QDeviance relative to that of the best fitting model (with the lowest QDeviance); Δ QAICc = QAICc relative to the best-supported model (with the lowest QAICc).  ^1^ QAIC_c_ = 6775,21  ^2^ QDev = 1467,45 | | | | | |

Table S3. Estimates and 95% confidence intervals of resighting probability for both sexes and different ring-types (codeflag or color combination) across (a) pre-fledging period, (b) post-fledging period and (c) adulthood. Estimates are based on model 1 (Table 3)

| a) | Year | Male codeflag | Female codeflag | Male color combination | Female color combination |
| --- | --- | --- | --- | --- | --- |
|  | 2008 | 0.67 (0.59–0.76) | 0.64 (0.56–0.73) | 0.75 (0.66–0.83) | 0.71 (0.63–0.80) |
|  | 2009 | 0.76 (0.70–0.82) | 0.73 (0.67–0.79) | 0.84 (0.78–0.89) | 0.81 (0.74–0.86) |
|  | 2010 | 0.82 (0.79–0.86) | 0.79 (0.75–0.83) | 0.89 (0.86–0.92) | 0.86 (0.82–0.89) |
|  | 2011 | 0.66 (0.52–083) | 0.63 (0.49–0.80) | 0.74 (0.58–0.90) | 0.70 (0.55–0.87) |
|  | 2012 | 0.83 (0.80–0.87) | 0.80 (0.76–0.84) | 0.90 (0.86–0.93) | 0.87 (0.83–0.91) |
|  | 2013 | 0.88 (0.86–0.90) | 0.85 (0.82–0.88) | 0.93 (0.91–0.95) | 0.91 (0.89–0.93) |
|  | 2014 | 0.92 (0.89–0.94) | 0.89 (0.87–0.91) | 0.96 (0.94–0.97) | 0.94 (0.92–0.96) |
|  | 2015 | 0.87 (0.82–0.92) | 0.84 (0.78–0.90) | 0.93 (0.88–0.96) | 0.90 (0.85–0.94) |
|  | 2016 | 0.92 (0.86–0.96) | 0.90 (0.83–0.95) | 0.96 (0.91–0.98) | 0.95 (0.89–0.98) |

| b) | Year | Codeflag | Color combination |
| --- | --- | --- | --- |
|  | 2009 | 0.09 (0.05–0.19) | 0.19 (0.09–0.34) |
|  | 2010 | 0.21 (0.13–0.32) | 0.37 (0.24–0.53) |
|  | 2011 | 0.32 (0.24–0.41) | 0.52 (0.42–0.63) |
|  | 2012 | 0.17 (0.06–0.39) | 0.31 (0.12–0.60) |
|  | 2013 | 0.21 (0.15–0.30) | 0.38 (0.27–0.50) |
|  | 2014 | 0.38 (0.32–0.45) | 0.59 (0.50–0.67) |
|  | 2015 | 0.35 (0.27–0.43) | 0.55 (0.45–0.64) |
|  | 2016 | 0.55 (0.36–0.73) | 0.74 (0.56–0.87) |
|  | 2017 | 0.31 (0.17–0.51) | 0.51 (0.31–0.71) |

| c) | Year | Male codeflag | Female codeflag | Male color combination | Female color combination |
| --- | --- | --- | --- | --- | --- |
|  | 2010 | 0.09 (0.03–0.23) | 0.06 (0.02–0.23) | 0.19 (0.07–0.42) | 0.14 (0.04–0.42) |
|  | 2011 | 0.13 (0.07–0.24) | 0.22 (0.12–0.37) | 0.27 (0.15–0.43) | 0.41 (0.25–0.58) |
|  | 2012 | 0.36 (0.26–0.47) | 0.29 (0.21–0.40) | 0.57 (0.46–0.68) | 0.50 (0.38–0.62) |
|  | 2013 | 0.48 (0.37–0.60) | 0.51 (0.39–0.63) | 0.69 (0.58–0.78) | 0.72 (0.60–0.81) |
|  | 2014 | 0.58 (0.48–0.67) | 0.47 (0.37–0.58) | 0.77 (0.68–0.83) | 0.68 (0.58–0.77) |
|  | 2015 | 0.68 (0.60–0.75) | 0.58 (0.47–0.67) | 0.84 (0.78–0.88) | 0.77 (0.68–0.83) |
|  | 2016 | 0.70 (0.62–0.77) | 0.55 (0.45–0.64) | 0.85 (0.79–0.89) | 0.74 (0.66–0.82) |
|  | 2017 | 0.69 (0.58–0.78) | 0.57 (0.43–0.69) | 0.84 (0.76–0.90) | 0.76 (0.65–0.84) |
